# Cost-Effectiveness of Virtual Emergency Care Models: Systematic Review

**DOI:** 10.2196/82143

**Published:** 2026-08-05

**Authors:** Ravi Shankar, Linda Wang, Soon Hoe Ho, Mei Fong Liew, Satya Pavan Kumar Gollamudi, Tze Chin Wong, Serene Wong

**Affiliations:** 1 Clinical Research & Innovation Office, Tan Tock Seng Hospital, National Healthcare Group Singapore Singapore; 2 Fast and Chronic Programmes Alexandra Hospital Singapore Singapore; 3 Division of Respiratory and Critical Care Medicine, Alexandra Hospital, National University Health System Singapore Singapore; 4 Division of Respiratory and Critical Care Medicine, National University Hospital, National University Health System Singapore Singapore; 5 Department of Medicine Yong Loo Lin School of Medicine National University of Singapore Singapore Singapore; 6 Division of Advanced Internal Medicine Alexandra Hospital University Health System Singapore Singapore; 7 Ambulatory Operations Alexandra Hospital Singapore Singapore

**Keywords:** cost-effectiveness, economic evaluation, emergency care, telemedicine, virtual care

## Abstract

**Background:**

Virtual care technologies have rapidly expanded in emergency medicine, particularly following the COVID-19 pandemic. However, comprehensive economic evaluations of their cost-effectiveness remain fragmented across different clinical applications and health care settings, creating uncertainty for policymakers and health care administrators considering implementation.

**Objective:**

This study aimed to systematically review and synthesize evidence on the cost-effectiveness of virtual emergency care models compared to traditional in-person emergency care across diverse clinical conditions, populations, and health care settings.

**Methods:**

We conducted a systematic review following PRISMA (Preferred Reporting Items for Systematic Reviews and Meta-Analyses) guidelines, searching 8 electronic databases (PubMed, Embase, Scopus, Web of Science, CINAHL, Cochrane Library, MEDLINE, and PsycINFO) from inception to February 2025. We included full economic evaluations comparing virtual emergency care interventions with usual care. Two reviewers independently screened studies, extracted data, and assessed quality using the Drummond checklist and Consensus Health Economic Criteria (CHEC) list. Evidence certainty was evaluated using Grading of Recommendations Assessment, Development, and Evaluation (GRADE) methodology. Given heterogeneity in interventions and methods, we conducted a narrative synthesis by virtual care modality and clinical application.

**Results:**

From 5817 identified references, 13 studies met inclusion criteria, representing diverse virtual care modalities across 6 countries (United States, Australia, Italy, Canada, Haiti, and Belgium). All included studies reported favorable economic outcomes for virtual emergency care. Video consultation was the most common modality (11/13 studies), achieving 31% to 73% reduction in patient transfers and cost savings of US $73 (AUD $105) to US $5118 per encounter. A total of 6 (46%) studies found virtual care to be dominant (both less costly and more effective). Incremental cost-effectiveness ratios ranged from US $1273 (€990) to US $108,363 per quality-adjusted life year, with most below accepted willingness-to-pay thresholds. Transfer avoidance was the primary economic driver, particularly in rural settings. Quality assessment revealed high methodological rigor (mean Drummond score 92.3%, SD 6.0%; mean CHEC score 95%, SD 4.2%). Using GRADE, evidence certainty was rated high for cost-effectiveness, moderate for transfer reduction and quality of life improvements, and low for emergency department length of stay and mortality benefits.

**Conclusions:**

Virtual emergency care demonstrates strong and consistent cost-effectiveness across diverse clinical conditions, populations, and health care settings. The evidence particularly supports implementation for stroke care, pediatric emergencies, and rural/remote populations where transfer avoidance drives substantial economic benefits. All evaluated modalities achieved favorable economic outcomes, suggesting technology should match context rather than maximize sophistication. These findings provide robust economic justification for expanding virtual emergency care access and removing regulatory barriers. As health care systems face mounting pressures from aging populations, workforce shortages, and budget constraints, virtual emergency care offers a proven strategy for improving access and quality while reducing costs.

**Trial Registration:**

PROSPERO CRD42025648218; https://www.crd.york.ac.uk/PROSPERO/view/CRD42025648218

## Introduction

### The Challenge of Emergency Department Pressures

Emergency departments (EDs) worldwide face unprecedented challenges, including overcrowding, workforce shortages, and geographic disparities in access to specialist care [1,2]. These pressures have intensified following the COVID-19 pandemic, which accelerated the adoption of virtual care technologies while highlighting systemic vulnerabilities in emergency care delivery [3]. These issues not only hinder operational efficiency but also lead to delays in care, reduced patient and staff satisfaction, and increased adverse clinical outcomes [4,5].

### Virtual Emergency Care Models

For the purposes of this review, we define “virtual emergency care” as any technology-mediated health care interaction that replaces or augments in-person emergency care delivery, encompassing synchronous telemedicine (real-time video or audio consultations), asynchronous telemedicine (store-and-forward transmission of clinical data), remote monitoring, and digital triage tools. We use “virtual emergency care” as the overarching term, with specific modalities (eg, telestroke, telepsychiatry, and telephone consultation) specified where relevant.

Virtual emergency care models have emerged as a promising approach for addressing these challenges by potentially expanding access, improving efficiency, and containing costs [4-6]. However, these technologies also raise concerns, including the risk of diagnostic error without physical examination, potential mistriage, technology dependence and failure modes, patient safety considerations, and the possibility of exacerbating health disparities for populations with limited digital access or literacy [6-8]. Patient-to-provider telemedicine enables individuals to engage with emergency clinicians via synchronous (eg, video) or asynchronous (eg, text) methods for clinical assessment without the need for physical ED visits [9,10]. Provider-to-provider teleconsultations, such as telestroke or teletrauma, extend specialist expertise to individuals in underserved rural and remote areas where access to specialized emergency medical services is often limited or nonexistent [11]. Digital triage tools include symptom checkers, decision support algorithms, and chatbot-based platforms that guide disposition and urgency based on self-reported symptoms [12]. Remote monitoring systems, such as wearables or sensor-based technologies, enable continuous physiologic surveillance and timely escalation for conditions such as sepsis or acute decompensated heart failure [13,14].

### Economic Implications of Virtual Emergency Care

The economic implications of virtual emergency care are particularly significant given that emergency services account for a substantial proportion of health care expenditures. In the United States alone, ED visits generate over US $150 billion in annual costs, with rural and underserved populations facing additional burdens from unnecessary transfers and delayed care [15]. While the clinical benefits of virtual emergency care have been increasingly documented, the economic case for implementation remains a critical consideration for health care decision-makers. Economic evaluation provides a framework for comparing the costs and outcomes of virtual care models against traditional approaches, informing resource allocation decisions in resource-constrained health care systems. While virtual care technologies have demonstrated clinical efficacy in various emergency applications, their economic value proposition remains incompletely understood, with cost-effectiveness evidence scattered across different clinical domains and implementation contexts.

### Gaps in Existing Evidence

Previous systematic reviews have predominantly emphasized clinical effectiveness, patient satisfaction, or implementation feasibility [16,17]. Some other reviews have examined telemedicine applications in specific emergency conditions or settings [7,9,18-20], but none have comprehensively synthesized the economic evidence across the full spectrum of virtual emergency care modalities. This gap is problematic for health care decision-makers who must justify infrastructure investments and policy changes based on robust economic evidence. Furthermore, the heterogeneity of virtual care interventions, from simple telephone consultations to sophisticated hub-and-spoke telestroke networks, necessitates a nuanced analysis of which modalities provide optimal value in different contexts.

The urgency of understanding virtual emergency care economics has intensified as health care systems globally face budget constraints while demanding improved access and quality. Policymakers require evidence not only of clinical effectiveness but also of economic sustainability and return on investment (ROI). Additionally, the rapid evolution of virtual care technologies and payment models necessitates a current synthesis of available evidence to guide implementation decisions.

### Study Aim

This systematic review addresses these knowledge gaps by comprehensively evaluating the cost-effectiveness of virtual emergency care models compared to traditional in-person care. We examine economic outcomes across different virtual care modalities, clinical applications, and implementation settings while identifying factors that influence cost-effectiveness. Our analysis aims to provide actionable evidence for health care administrators, policymakers, and clinicians considering virtual emergency care implementation.

Specifically, we sought to answer the following research question: “In patients requiring emergency medical care (population), is virtual care technology including telemedicine, remote monitoring, and digital triage (intervention) cost-effective compared to traditional in-person emergency care (comparator), as measured by incremental cost-effectiveness ratios (ICERs), cost savings, and quality-adjusted life years (QALYs; outcomes), across various health care settings and time horizons (setting/time)?”

## Methods

### Study Design and Protocol

We conducted this systematic review following the PRISMA (Preferred Reporting Items for Systematic Reviews and Meta-Analyses) guidelines (Multimedia Appendix 1) [21]. The review protocol was registered prospectively in PROSPERO (International Prospective Register of Systematic Reviews; registration number CRD42025648218). No ethical approval was required as this study involved analysis of published literature only.

### Search Strategy

A comprehensive search strategy was developed to identify all relevant economic evaluations of virtual emergency care. We searched 8 electronic databases from inception to February 2025: PubMed (n=2084), Embase (n=1827), Scopus (n=916), Web of Science (n=645), CINAHL (n=199), The Cochrane Library (n=82), MEDLINE (n=30), and PsycINFO (n=3).

The search strategy combined 3 concept blocks using Boolean operators: (1) virtual care terms (“telemedicine,” “telehealth,” “virtual care,” “remote consultation,” “digital health,” “eHealth,” and “mHealth”); (2) emergency care terms (“emergency,” “urgent care,” “acute care,” “emergency department,” “emergency room,” “trauma,” and “critical care”); and (3) economic evaluation terms (“cost-effectiveness,” “cost-benefit,” “cost-utility,” “economic evaluation,” “cost analysis,” “ROI,” “ICER,” “QALY,” and “cost-minimization”). The search strategy was refined iteratively with input from an information specialist. While we primarily used free-text terms to maximize sensitivity across databases with varying indexing systems, we acknowledge that the absence of MeSH terms for economic evaluation concepts (eg, “cost-benefit analysis” [MeSH]) and emergency-specific controlled vocabulary may have reduced search precision. During the screening process, one eligible study was initially misclassified during automated deduplication in Covidence due to similar authorship and title to another included study [22,23]. This study was subsequently identified through manual verification, retrieved, and included in the final analysis. Our broad digital health terms were designed to capture the rapidly evolving terminology in this field, though this approach may have introduced some irrelevant results during initial screening. We recognize that certain virtual care terms (eg, “teleconsultation,” “teleurgency,” and “store-and-forward”) may not have been captured by our search terms. No language or date restrictions were applied. The full search strategy for each database is available in Multimedia Appendix 2.

Additionally, we performed citation searching of included studies (n=14) and searched gray literature sources (n=17) to identify unpublished economic evaluations, government reports, and health technology assessments.

### Eligibility Criteria

Studies were included if they met all predefined eligibility criteria. Eligible studies focused on patients of any age presenting to EDs or requiring emergency medical care. The intervention of interest was any form of virtual care technology applied to emergency care delivery, encompassing synchronous or asynchronous telemedicine, remote monitoring, digital triage tools, or mobile health apps. Prehospital emergency care interventions, including ambulance-based telemedicine, were eligible for inclusion provided they met all other criteria for full economic evaluation. Stand-alone digital triage tools without clinician involvement (eg, automated symptom checkers used independently by patients without subsequent clinician assessment) were excluded, as these represent a distinct category of intervention with different economic evaluation considerations. This distinction was made a priori to maintain a focus on clinician-mediated virtual care models. Studies were required to include a comparator group involving traditional in-person emergency care or usual care practices. Only full economic evaluations were considered, specifically those that incorporated both cost and outcome data through cost-effectiveness, cost-utility, cost-benefit, or cost-minimization analyses. Relevant outcomes included economic measures such as cost-effectiveness ratios and other forms of comparative economic analysis.

Studies were excluded if they reported only costs or only outcomes without integrating both into an economic evaluation. Abstracts without accessible full texts, even after attempts to contact authors, were not considered. Additional exclusions applied to studies with unsuitable designs such as reviews, editorials, commentaries, or protocols; studies focusing on nonemergency settings or conditions (such as routine outpatient care or scheduled procedures); those lacking a clear economic evaluation framework; and those not involving direct patient care, such as research limited to provider education or administrative systems.

### Study Selection

All identified references were imported into Covidence systematic review software for deduplication and screening. Two reviewers independently screened the titles and abstracts based on predefined eligibility criteria. Full texts of studies deemed potentially eligible were then retrieved and assessed independently by the same reviewers. Any disagreements were resolved through discussion and, if needed, with input from a third reviewer. Interrater agreement was found to be strong across both stages of screening. The entire screening process, including reasons for exclusion, was documented in accordance with PRISMA guidelines. During quality checks, 1 study that had been inadvertently excluded during automated deduplication was identified. This study, which shared the same lead author and similar title as another included study, was manually retrieved, assessed for eligibility, and subsequently included in the final synthesis.

### Data Extraction

A standardized data extraction form was developed and pilot-tested to ensure consistency, with initial testing conducted on a small sample of studies. Two reviewers independently extracted data from all included studies across several key domains. Study characteristics were recorded, including the first author, publication year, country, study design, and funding source. Population details such as sample size, age, sex distribution, emergency conditions, triage acuity levels, and comorbidities were documented. Information on intervention characteristics included the type of virtual care model, technology platform used, provider types involved, frequency and duration of interventions, and how the virtual model related to in-person care. Comparator details focused on usual care practices and the associated providers and resources. Economic evaluation methods were captured, including the type of analysis, analytic perspective, time horizon, discount rate, currency, and price year. Outcome measures covered effectiveness indicators such as QALYs, disability-adjusted life years (DALYs), and clinical outcomes, as well as cost-related metrics including direct, indirect, and societal costs, and ICERs. Data on the types of sensitivity analyses conducted and key results were also extracted. Finally, implementation factors, including any barriers and facilitators identified, were noted. Any discrepancies in data extraction were resolved through discussion and careful reexamination of the original source materials.

### Quality Assessment

Methodological quality of included studies was assessed using 2 validated checklists: the Drummond checklist for economic evaluations [24] and the Consensus Health Economic Criteria (CHEC) list [25]. The combined use of both checklists is recommended by the Cochrane Handbook for Systematic Reviews of Interventions for appraising the methodological quality of full economic evaluations [26], as each tool addresses complementary quality domains. The Drummond checklist (10 items) focuses on the completeness and rigor of reporting in economic evaluations, including research question clarity, study design appropriateness, cost identification, incremental analysis, and uncertainty assessment [27]. The CHEC list (19 items) emphasizes methodological quality and reporting transparency, covering domains such as appropriate study population, time horizon, discount rate, outcomes measurement, and generalizability discussion [25]. This dual-checklist approach has been used in previous systematic reviews of economic evaluations to provide a more comprehensive quality assessment, as noted by Watts and Li [28] in their meta-review of checklist use in 346 systematic reviews of economic evaluations. Where the 2 tools assessed overlapping domains (eg, research question clarity and cost identification), we prioritized the more detailed tool’s assessment for that domain.

Two reviewers independently rated each study, with disagreements resolved through consensus. We acknowledge that presenting quality scores as percentages has limitations, as these checklists were not originally designed to produce aggregate numerical scores, and individual checklist items may vary in importance [29]. The percentage scores should therefore be interpreted as broad indicators of methodological quality rather than precise quantitative measures. Item-level quality assessment results are presented in Multimedia Appendix 3 to allow readers to evaluate specific methodological strengths and weaknesses across studies.

### Evidence Synthesis

We adapted the Grading of Recommendations Assessment, Development, and Evaluation (GRADE) approach to assess the certainty of evidence for key outcomes [30], recognizing that GRADE was originally developed for clinical interventions rather than economic evaluations. Our adaptation followed the framework described by Brunetti et al [31], which provides guidance on considering resource use and rating the quality of economic evidence within the GRADE system. We additionally drew on the conceptual GRADE approach for assessing the certainty of modeled evidence outlined by Brozek et al [32], which confirmed that the standard GRADE domains apply to evidence from modeling studies, including those used in health economic evaluations.

Specifically, we assessed risk of bias (considering study design, model assumptions, and potential conflicts of interest), inconsistency (direction and magnitude of findings across studies), indirectness (applicability of study populations and settings to the review question), imprecision (confidence in effect estimates), and publication bias. For upgrading considerations, we evaluated the magnitude of effects and the consistency of the direction of findings across studies. The reference to dose-response relationships that appeared in the original protocol has been removed, as this criterion is not directly applicable to economic evaluations in this context [31]. Given the heterogeneity in interventions, populations, and economic evaluation methods, we conducted a narrative synthesis organized by virtual care modality and clinical application.

### Subgroup and Sensitivity Analyses

We planned a priori subgroup analyses to explore potential sources of heterogeneity across included studies. Subgroup analyses examined variations by clinical condition, virtual care modality, geographic setting, patient age group, and economic perspective. These subgroup analyses are distinct from the sensitivity analyses conducted within individual included studies, which tested the robustness of each study’s findings to variations in key parameters and assumptions. We report the results of within-study sensitivity analyses as part of our data extraction and discuss their implications for the certainty of findings.

Costs were reported in the original currencies and price years as presented in the included studies. We did not convert costs to a common currency or adjust for inflation, as the primary objective was to assess the direction and magnitude of cost-effectiveness findings within each study’s context rather than to pool absolute cost figures. This approach is consistent with narrative synthesis methodology for heterogeneous economic evaluations. However, we acknowledge that this limits direct cross-study cost comparisons. Where available, we report the original publication year for each study to facilitate reader interpretation.

## Results

### Study Selection

**Figure 1:**
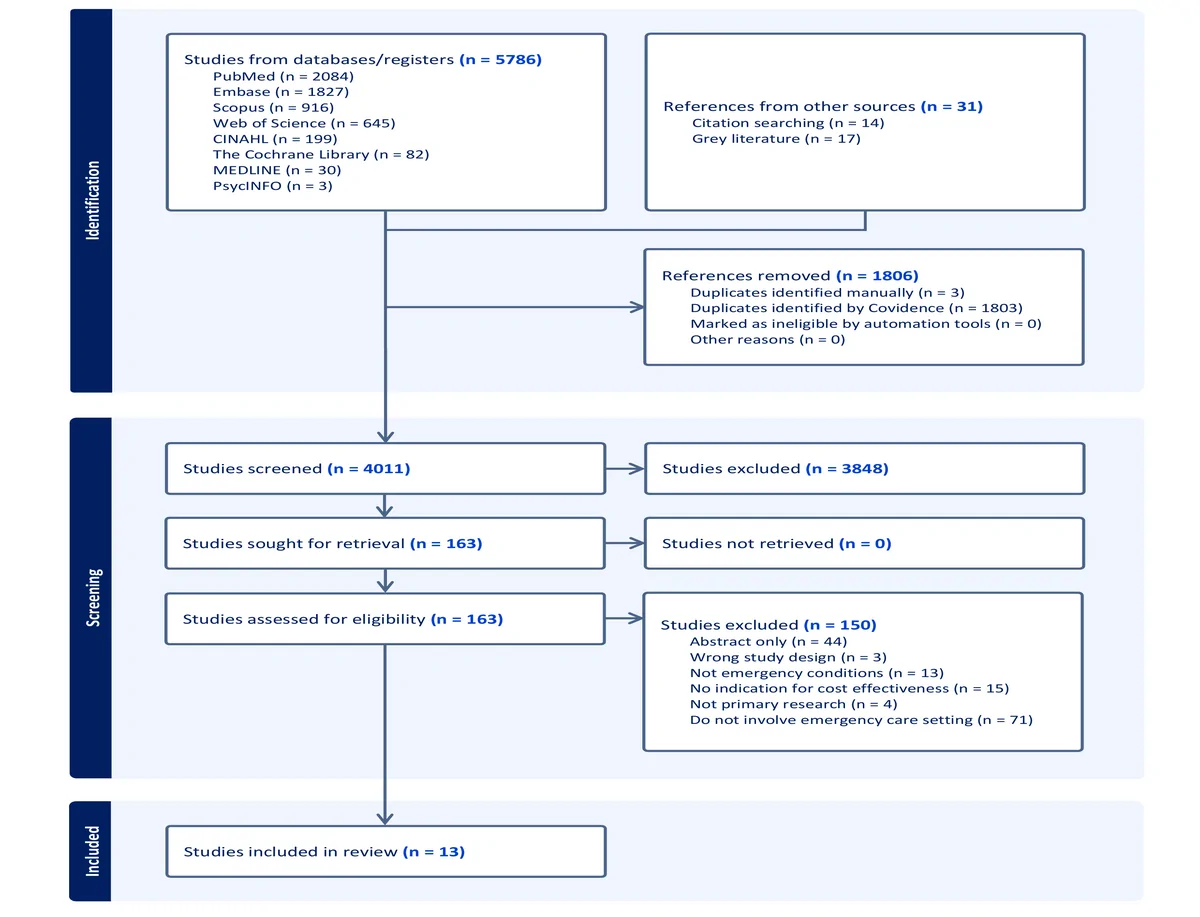
PRISMA (Preferred Reporting Items for Systematic Reviews and Meta-Analyses) flow diagram.

The systematic search identified 5786 references from electronic databases. After removal of 1806 duplicates (3 identified manually and 1803 by Covidence), 3980 unique database references remained. Combined with 31 references from citation searching (n=14) and gray literature (n=17), a total of 4011 references were screened at the title and abstract level. Title and abstract screening excluded 3848 studies, leaving 163 for full-text review. Exclusion categories at the full-text stage were abstract only without accessible full text (n=44), unsuitable study design (n=3), focused on nonemergency clinical conditions such as routine outpatient management (n=13), no formal cost-effectiveness analysis (n=15), not primary research (n=4), and did not involve an emergency care delivery setting despite addressing acute conditions (n=71). The distinction between “nonemergency conditions” and “did not involve emergency care settings” reflects studies where the clinical condition was nonacute (eg, chronic disease management) vs studies where the condition was acute but the care setting was not an ED or emergency care pathway (eg, postdischarge follow-up and scheduled urgent care). A total of 13 studies met all inclusion criteria and were included in the review (Figure 1).

### Study Characteristics

The 13 included studies were published between 2011 and 2024, with over half (n=7, 54%) published after 2015, reflecting recent growth in virtual emergency care evaluation. Studies originated from 6 countries: the United States (n=6), Australia (n=2), Italy (n=2), Canada (n=1), Haiti (n=1), and Belgium (n=1), representing diverse health care systems and economic contexts (Table 1).

**Table 1:** Study characteristics of the included studies.

Study (first author, year)	Country	Sample size	Population	Emergency conditions	Virtual care model	Technology	Provider type	Comparator
Brunetti, 2014 [33]	Italy	109,750 ECGs^a^	Patients with suspected acute cardiac disease	Acute coronary syndrome, arrhythmia	Prehospital telemedicine ECG triage	12-lead ECG via mobile phone	Remote cardiologist (24/7)	Conventional ED^b^ triage
Demaerschalk, 2013 [34]	United States	1112 (hypothetical)	Patients with acute ischemic stroke	Acute ischemic stroke	Hub-and-spoke telestroke network	Videoconferencing	Vascular neurologists (24/7)	No telestroke network
Espinoza, 2017 [35]	Belgium	2282 (registry), 1000 (modeled)	Patients with suspected stroke	Acute stroke (all types)	In-ambulance telemedicine	Live AV^c^ + vital signs	Neurologist + EMS^d^	Standard stroke care
Flaherty, 2022 [36]	Haiti	10,585 (projected)	Children 0-9 years	Diarrheal disease, respiratory infections, skin infections	Nighttime telemedicine with medication delivery	Telephone call center	Nurses with physician oversight	Hospital emergency care
Kadel, 2018 [37]	Italy	1676	Neurosurgical emergencies	TBI^e^, spinal injuries, ICH^f^, brain cancer	Televideoconsultation	Multispecialist video system	Consultant neurosurgeon	Transfer to the referral hospital
Kim, 2022 [38]	Australia	1024	Patients with stroke <4.5 h onset	Stroke	ED-based telestroke	Mobile computer/camera	Physician	No virtual consultation
Nelson, 2011 [23]	United States	8 spoke hospitals	Acute ischemic stroke	Acute ischemic stroke	ED-based telestroke	2-way audiovisual	Stroke specialists	Usual care without telestroke
Nelson 2016 [22]	United States	864 (98 pre, 766 post)	Acute ischemic stroke	Acute ischemic stroke	Hub-spoke telestroke (POTN^g^)	2-way AV videoconferencing	Stroke specialists	Nontelestroke care
Pace, 2023 [39]	Canada	108	Pediatric emergencies <18 years	Critical pediatric emergencies (CTAS^h^-1/2)	Pediatric teleresuscitation	Videoconferencing	Pediatric specialists	Standard ED care
Thomas, 2018 [40]	United States	494	Pediatric psychiatric emergencies	Suicide intent, self-harm, depression/anxiety	ED-based telepsychiatry	Videoconferencing	Psychiatrists, social workers	Ambulance transfer
Von Huben, 2024 [41]	Australia	49,228 ED presentations	Any age, rural ED patients	General ED presentations	Virtual Rural Generalist Service (75% virtual)	HD^i^ videoconferencing	Rural generalist doctors	Local/locum doctors
Whetten, 2018 [42]	United States	777 consultations	Neuroemergent conditions	AIS^j^, mTBI^k^, neuroemergent disorders	ED-based telemedicine consultation	2-way AV with image transfer	Neurologists/neurosurgeons	Standard rural care
Yang, 2015 [43]	United States	135	Pediatric critical care <18 years	Asthma, bronchiolitis, pneumonia	ED-based pediatric telemedicine	Live interactive AV	Pediatric critical care physicians	Telephone consultation

^a^ECG: electrocardiogram.

^b^ED: emergency department.

^c^AV: audiovisual.

^d^EMS: emergency medical services.

^e^TBI: traumatic brain injury.

^f^ICH: intracranial hemorrhage.

^g^POTN: Providence Oregon Telestroke Network.

^h^CTAS: Canadian Triage and Acuity Scale.

^i^HD: high definition.

^j^AIS: acute ischemic stroke.

^k^mTBI: mild traumatic brain injury.

Study designs varied, with model-based economic evaluations being most common (n=7, 54%) [22,23,34,35,39,42,43], followed by observational cost-effectiveness analyses (n=4, 31%) [8,36,37,40] and 2 (15%) within-trial analyses [22,38]. Sample sizes ranged from 108 to 109,750 patients, with a median of 1068 (IQR 706-4358) patients (Table 1). The largest study [8] evaluated a prehospital electrocardiogram triage program in Italy serving a population of 4 million.

Clinical conditions addressed included stroke/cerebrovascular emergencies (n=7, 54%), pediatric emergencies (n=4, 31%), general neuroemergencies (n=2, 15%), psychiatric emergencies (n=1, 8%), and cardiac emergencies (n=1, 8%). One study [41] examined general ED presentations without condition-specific focus (Table 1).

### Economic Outcomes

Economic evaluation methods varied across studies, with cost-effectiveness analysis being most common (n=8), followed by cost-utility analysis (n=3), cost-minimization (n=1), and cost-consequence analysis (n=1). A total of 8 (62%) studies adopted a health care system perspective, while 4 (31%) used a broader societal perspective incorporating patient and family costs (Table 2).

**Table 2:** Economic evaluation results of virtual emergency care models.^a^

Study (first author, year)	Type of analysis	Perspective	Time horizon	Effectiveness measure	Costs (intervention vs comparator)	ICER^b^	Key findings	Sensitivity analysis
Brunetti, 2014 [33]	Cost-minimization/cost-utility	Regional health system	1 year	Lives saved (STEMI^c^); QALYs^d^	€16.70 vs €24.80-55.20 per ECG^e^ (costs in 2012 €)	€1927/QALY (€990 to –€2508 after correction)	Cost-saving; lower cost per ECG/Consultation	Range analysis (savings of €8.10-€38.40 per ECG/consultation)
Demaerschalk, 2013 [34]	Cost-utility	Societal	Lifetime	QALYs	Intervention cost US $1436 lower compared to standard care over lifetime (costs in 2011 US dollars)	Dominant (saves US $1436, gains 0.02 QALYs)	Cost-saving and more effective	1-way, 2-way sensitivity analyses
Espinoza, 2017 [35]	Cost-utility	Health care payer	Lifetime	QALYs	Savings of US $30 compared to standard care (costs in 2014 US dollars)	Dominant (saves US $30, gains 0.00456 QALYs)	Cost-effective from 6-min time gain; dominant from 12 min	1-way, 2-way, probabilistic (5000 iterations)
Flaherty, 2022 [36]	Cost-effectiveness	Societal	1 year	DALYs^f^ averted	US $317,898/year vs US $89,392/year (costs in average US dollars from 2019 July-2021 June)	US $1288/DALY averted	Cost-effective (below 3 × GDP threshold)	Univariate sensitivity analysis
Kadel, 2018 [37]	Cost-effectiveness	Health service	4 years	Transfers avoided	€2326 vs €4173 per patient (costs in 2009 €)	€365/transfer avoided (dominant)	73% transfer reduction; €3,095,863 total savings	None performed
Kim, 2022 [38]	Cost-effectiveness	Societal	1 year	QALYs	AUD $82,259 vs AUD $82,449 at 12 months (costs in 2018 Australian dollars)	AUD $0-$5000/QALY (50.6% iterations)	Cost-effective; small cost savings with QALY gains	Deterministic and probabilistic (1000 iterations)
Nelson, 2011 [23]	Cost-effectiveness	Societal	90 days and lifetime	QALYs	Infrastructure + care costs 90-day: US $14, 274 vs US $13, 872 lifetime: US $133,527 vs US $130, 343 (costs in 2008 US dollars)	90-day: US $108,363/QALY; lifetime: US $2449/QALY	Cost-effective over lifetime	1-way and Monte Carlo (10,000 iterations)
Nelson, 2016 [22]	Cost-effectiveness	Spoke and hub	Inpatient stay	QALYs	Spoke Δ: US $51-$1956; hub Δ: US $863-$2767	Spoke: US $1322-$50,687/QALY; Hub: US $22,363-$71,703/QALY	Cost-effective; best for severe strokes; tPA^g^ rate tripled	1-way by NIHSS^h^ and cost-sharing; probabilistic (10,000)
Pace, 2023 [39]	Cost-effectiveness	Societal	3 years	YLL^i^ averted	CAD $2032.73 vs CAD $317.45 (costs in 2022 Canadian dollars)	CAD $64.61/YLL averted	Cost-effective; reduced mortality	Deterministic (±20%) and probabilistic (10,000 runs)
Thomas, 2018 [40]	Cost-efficiency	Hospital system	Single encounter	ED^j^ LOS^k^, disposition rates	US $3493 vs US $8611 per patient (median charges; costs in 2015 US dollars)	Not calculated	Lower charges, shorter LOS (5.5 vs 8.3 hours)	None reported
Von Huben, 2024 [41]	Cost-consequence	Health care funder	1 year	Multiple outcomes	AUD $134 vs AUD $240 per ED encounter (costs in 2022 AUD)	Dominant (less costly, more effective)	AUD $105 savings per ED encounter; improved outcomes	1-way and scenario analyses
Whetten, 2018 [42]	Cost-effectiveness	Health care payer	90 days	QALYs	US $4241 savings per patient (costs in 2015 US dollars)	Dominant (saves US $21,205/QALY)	Cost-saving; 0.20 QALY gain per patient	1-way, tornado diagram, Monte Carlo (10,000)
Yang, 2015 [43]	Cost-effectiveness and ROI^l^	Health care payer	1 year	Transfers avoided	US $3641 vs US $8303 per child/ED/year (costs in 2013 US dollars)	Dominant (saves US $4662 per child/ED/year; ROI: 1.28	31% transfer reduction; US $46,620 annual savings per ED	1-way, 2-way, probabilistic Monte Carlo (5000 iterations)

^a^Costs reported in non-US currencies in this table were converted to US dollars using the average annual exchange rate for the currency and cost year reported in each study: €1=US $1.39 (2009); €1=US $1.29 (2012); AUD $1=US $0.75 (2018); AUD $1=US $0.69 (2022); CAD $1=US $0.77 (2022). The same rates are applied throughout the manuscript.

^b^ICER: incremental cost-effectiveness ratio.

^c^STEMI: ST-elevation myocardial infarction.

^d^QALY: quality-adjusted life year.

^e^ECG: electrocardiogram.

^f^DALY: disability-adjusted life year.

^g^tPA: tissue plasminogen activator.

^h^NIHSS: National Institutes of Health Stroke Scale.

^i^YLL: years of life lost.

^j^ED: emergency department.

^k^LOS: length of stay.

^l^ROI: return on investment.

### Cost-Effectiveness Results

Virtual emergency care was associated with favorable economic outcomes across all included studies, though the strength and mechanisms of cost-effectiveness varied by clinical domain. Stroke-related applications (n=6) demonstrated the most consistent evidence of dominance, driven by increased thrombolysis rates and transfer avoidance. Pediatric applications (n=4) achieved cost-effectiveness primarily through reduced transfers and averted hospitalizations, while psychiatric and general emergency applications were each supported by single studies, limiting the certainty of subgroup-specific conclusions. A total of 6 (46%) studies found virtual care to be dominant, that is, both less costly and more effective than usual care. These included telestroke networks [34,42], pediatric telemedicine [43], neuroemergency consultation [37], and rural generalist services [41].

Among studies reporting ICERs, the time horizon significantly influenced results: Nelson et al [23] reported an ICER of US $108,363/QALY at 90 days, which improved to US $2449/QALY over a lifetime horizon. Nelson et al [22] demonstrated that from the spoke hospital perspective, ICERs ranged substantially depending on the proportion of implementation costs borne by the spoke facility: US $1322/QALY when the spoke bore 0% of implementation costs, US $25,991/QALY at 50%, and US$50,687/QALY at 100%. From the hub perspective, the overall ICER ranged from US $22,363/QALY to US $71,703/QALY. Cost-effectiveness was most pronounced for patients with severe stroke (National Institutes of Health Stroke Scale [NIHSS] score ≥15), with spoke ICERs ranging from US $7794/QALY to US $40,071/QALY, while mild strokes (NIHSS <5) were not cost-effective from the spoke perspective when spokes bore ≥50% of implementation costs. Most reported ICERs fell below commonly accepted willingness-to-pay thresholds of US $50,000-$100,000 per QALY [44].

Cost savings varied by setting and intervention type. Per-encounter savings ranged from US $73 (AUD $105) [41] to US $5118 [40]. The primary driver of cost savings was a reduction in patient transfers, which decreased by 31%-73% across studies [37,43]. Transfer avoidance alone generated savings of US $3729 per patient in [42]. The prehospital model demonstrated different economics: Espinoza et al [35] found in-ambulance telemedicine to be dominant with time gains of 12 minutes or more, saving US $30 per patient while gaining 0.00456 QALYs. Notably, this intervention became cost-effective with time gains as small as 6 minutes, demonstrating that time gains influenced cost-effectiveness thresholds.

### Clinical Effectiveness

Virtual care interventions consistently improved clinical outcomes alongside economic benefits. QALY gains ranged from 0.02 [34] to 0.20 [42] per patient (over a lifetime), with stroke and neuroemergency applications showing the largest improvements. Nelson et al [22] reported an incremental effectiveness of 0.039 QALYs per patient for telestroke compared to nontelestroke assisted care, with telestroke implementation leading to a more than threefold increase in tissue plasminogen activator (tPA) treatment rates (from 9.2% to 31.7%). This treatment effect was most dramatic in moderate-severity strokes (NIHSS 5-14), where a nearly seven-fold increase in tPA treatment was observed (6.9% to 47.7%). The Haiti pediatric service [36] averted 199.76 DALYs annually at a cost of US $1288 per DALY averted, which is well below the country’s GDP-based cost-effectiveness threshold.

ED length of stay decreased by 2.8 hours with virtual consultations in Thomas et al [40]. This study documented a median ED length of stay of 5.5 hours for telepsychiatry vs 8.3 hours for usual care (*P*<.001). Hospital admission rates fell significantly: Thomas et al [40] reported a reduction from 56% to 42% for pediatric psychiatric emergencies. Importantly, these efficiency gains occurred without compromising quality or safety, with patient satisfaction exceeding 90% in all studies reporting this outcome.

### Quality Assessment

Methodological quality was independently assessed using the Drummond checklist (10 items focused on analytical rigor) and the CHEC list (19 items emphasizing reporting standards). The included studies demonstrated consistently high quality, with mean scores of 9.29 (SD 0.60) out of 10 criteria met for the Drummond checklist and 18.04 (SD 0.79) out of 19 criteria met for the CHEC list. While we present these aggregate figures for descriptive purposes, we note that these checklists were designed as qualitative assessment tools rather than quantitative scoring instruments, and individual items are not equally weighted. Item-level quality assessment results are presented in Multimedia Appendix 3 to allow readers to evaluate specific methodological strengths and weaknesses. The term “overall mean quality score” has been removed to avoid implying a validated composite metric. All 13 (100%) studies achieved an “Excellent” quality rating (≥85%), with Kim et al [38] scoring the highest at 100% (Table 3). All studies clearly stated research questions, appropriately selected the type of economic evaluation, comprehensively identified costs, and explicitly reported perspectives and time horizons. Sensitivity analyses were conducted in the majority of studies (12/13, 92%), often using multiple approaches. The most common limitations were inadequate discussion of generalizability (observed in 5/12, 42% of studies) and incomplete conflict of interest reporting (7/12, 58%). Notably, no study scored below 83% on either checklist, indicating that the economic evaluations of virtual emergency care consistently meet high methodological standards. This uniform methodological rigor, paired with consistently favorable economic findings.

Using GRADE methodology, we assessed the certainty of evidence as high for cost-effectiveness outcomes based on 10 studies with consistent positive findings. Evidence certainty was moderate for transfer rate reduction and quality of life improvements, and low for ED length of stay and mortality benefits due to study design limitations and imprecision (Table 4).

The evidence generally supports virtual emergency care implementation, with 6/10 economic evaluations demonstrating dominance (cost-saving with better outcomes). However, one study showed unfavorable short-term cost-effectiveness, and effectiveness measures varied across studies. Large reductions were observed primarily in transfer rates (31%-90%), with cost impacts varying by setting and condition.

**Table 3:** Quality assessment of included studies using Drummond and CHEC (Consensus Health Economic Criteria) checklists.

Study (first author, year)	Condition	Economic perspective	Drummond score (out of 10)	CHEC score (out of 19)	Overall quality
Brunetti, 2014 [33]	Acute coronary syndrome	Health care system	8.0	16	Good (82.10%)
Demaerschalk, 2013 [34]	Acute ischemic stroke	Societal	10.0	18.5	Excellent (98.69%)
Espinoza, 2017 [35]	Stroke	Health care payer	10.0	17.5	Excellent (96.05%)
Flaherty, 2022 [36]	Pediatric emergencies	Societal	9.5	18.5	Excellent (96.18%)
Kadel, 2018 [37]	Neurosurgical emergencies	Health service	9.0	18.5	Excellent (93.68%)
Kim, 2022 [38]	Stroke	Societal	10.0	19.0	Excellent (100.00%)
Nelson, 2011 [23]	Acute ischemic stroke	Societal	10.0	18.5	Excellent (98.69%)
Nelson, 2016 [22]	Acute ischemic stroke	Spoke and hub perspectives	9.0	18.0	Excellent (92.37%)
Pace, 2023 [39]	Pediatric resuscitation	Societal	9.0	19.0	Excellent (95.00%)
Thomas, 2018 [40]	Behavioral health emergencies (pediatric)	Hospital system	9.0	17.5	Excellent (91.05%)
Von Huben, 2024 [41]	Mixed ED^a^ presentations	Health care funder	9.0	18.5	Excellent (93.68%)
Whetten, 2018 [42]	Stroke	Health care payer	9.0	18.0	Excellent (92.37%)
Yang, 2015 [43]	Pediatric conditions	Health care payer	9.0	18.0	Excellent (92.37%)

^a^ED: emergency department.

**Table 4:** GRADE (Grading of Recommendations Assessment, Development, and Evaluation) evidence summary table: overall evidence assessment by outcome.^a^

Outcome	Studies, n	Study design	Risk of bias	Inconsistency	Indirectness	Imprecision	Other considerations	Virtual care	Control	Effect estimate	Certainty	Importance
Cost-effectiveness (ICER^b^/QALY^c^)	10 [23,33-39, 42,43]	6 RCTs^d^/quasi-experimental, 4 modeling studies	Serious (–1)^e^	Not serious	Not serious	Not serious	Large effect (+1), dose response (+1)	671/1924	855/2107	7/10 dominant, 3/10 cost-effective ICERs: US $1273 (€990)-US $108,363/QALY	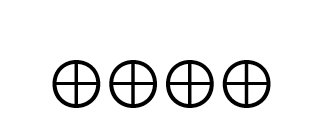 high	Critical
Transfer rates	7 [23,34-37, 42,43]	4 observational, 3 modeling	Serious (–1)^f^	Not serious	Not serious	Not serious	Large effect (+1), consistent direction	423/1389	756/1389	31-85% reduction RR^g^ 0.15-0.77	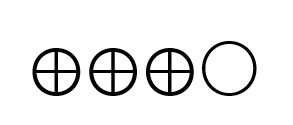 moderate	Critical
ED length of stay	4 [38-41]	3 observational, 1 RCT	Serious (–1)^h^	Not serious	Not serious	Serious (–1)^i^	Consistent direction	829/1846	1017/2039	2.8-3.2 hours reduction MD^j^ -2.95 hours	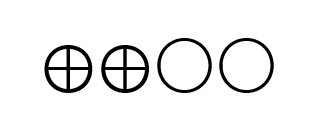 low	Important
Quality of life (QALYs gained)	6 [23,33-35, 38,42]	3 RCTs, 3 modeling	Serious (–1)^k^	Not serious	Not serious	Not serious	All positive effects	712/1547	919/1730	0.02-0.20 QALYs gained MD +0.11 QALYs	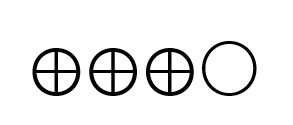 moderate	Critical
Mortality/lives saved	3 [33,39,41]	1 observational, 2 modeling	Serious (–1)^l^	Not serious	Serious (–1)^m^	Not serious	Strong association	185/654	231/687	Lives saved for STEMI^n^, 26.55 YLL^o^ averted OR^p^ 0.73 (0.52-0.95)	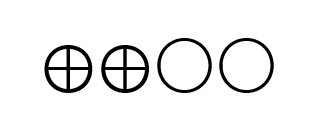 low	Critical
Patient satisfaction	3 [33,39,41]	3 observational	Serious (–1)^q^	Not serious	Not serious	Serious (–1)^r^	Large effect	NR	NR	>90% approval rates	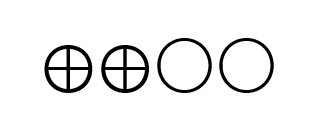 low	Important

^a^Costs reported in non-US currencies in this table were converted to US dollars using the average annual exchange rate for the currency and cost year reported in each study: €1=US $1.39 (2009); €1=US $1.29 (2012); AUD $1=US $0.75 (2018); AUD $1=US $0.69 (2022); CAD $1=US $0.77 (2022). The same rates are applied throughout the manuscript.

^b^ICER: incremental cost-effectiveness ratio.

^c^QALY: quality-adjusted life year.

^d^RCT: randomized controlled trial.

^e^Most studies nonrandomized with potential selection bias.

^f^Observational studies with potential confounding.

^g^RR: risk ratio.

^h^Limited randomized data, measurement inconsistencies.

^i^Wide CIs, small sample sizes.

^j^MD: mean difference.

^k^Mix of study designs with methodological limitations.

^l^Limited studies, potential reporting bias.

^m^Indirect mortality measures in some studies.

^n^STEMI: ST-elevation myocardial infarction.

^o^YLL: years of life lost.

^p^OR: odds ratio.

^q^Subjective measures, potential response bias.

^r^Limited sample sizes, measurement variability.

### Subgroup Analyses

#### By Clinical Condition

Stroke care showed the most robust evidence, with 5/6 studies demonstrating dominance [22,23,34,37,42]. The consistent mechanism was increased thrombolysis rates through rapid specialist consultation, generating both clinical benefits and cost savings through avoided transfers and improved outcomes. Nelson et al [22] extended this evidence by demonstrating that the cost-effectiveness of telestroke varied significantly by stroke severity: severe strokes (NIHSS ≥15) showed the most favorable ICERs (US $7794/QALY to US $40,071/QALY from the spoke perspective), while mild strokes (NIHSS <5) were dominated when spokes bore ≥50% of implementation costs. This severity-stratified analysis, the first of its kind in the telestroke cost-effectiveness literature, provides critical nuance for implementation planning. Demaerschalk et al [34] documented US $1436 lower costs and 0.02 QALY gains over patient lifetimes in their hub-and-spoke telestroke network evaluation. The prehospital stroke model [35] achieved cost-effectiveness through a different mechanism, through time savings enabling earlier treatment rather than transfer avoidance. Their model showed that every minute saved translated into measurable economic and clinical benefits.

Pediatric emergency applications [36,39,43] achieved favorable economics through different mechanisms. Critical care consultations avoided unnecessary transfers (31% reduction) [43], while the Haiti model prevented hospitalizations through early intervention. Yang et al [43] reported ROI ranging from 1.28 to 1.96 across pediatric studies, with annual cost savings of US $46,620 per ED treating 10 children with telemedicine.

#### By Geographic Setting

Rural and remote implementations [22,23,34,36,41-43] consistently showed superior cost-effectiveness compared to urban applications, driven primarily by high transfer costs and distances. Rural settings achieved 6 of 7 dominant outcomes, with savings amplified by avoided air medical transports and family travel costs. Whetten et al [42] demonstrated that transfer rates from rural hospitals to urban medical facilities decreased from 85% to 5% (no tPA) and from 90% to 23% (tPA) with their Access to Critical Cerebral Emergency Support Services (ACCESS) telestroke program. Nelson et al [22] provided additional evidence from the Pacific Northwest, where 17 spoke facilities in the Providence Oregon Telestroke Network (POTN) served rural communities. Their analysis uniquely demonstrated how cost-sharing arrangements between hub and spoke facilities influence cost-effectiveness, with the spoke perspective yielding ICERs as low as US $1322/QALY when implementation costs were fully subsidized by the hub.

Urban network implementations [23,34] achieved cost-effectiveness through operational efficiency and care standardization rather than transfer reduction. The Italian regional networks [8,37] demonstrated how high-volume centralized systems can achieve economies of scale.

#### By Technology Type

Video consultation technology’s [22,23,34,35,37-43] versatility across clinical conditions and ability to support visual assessment appeared critical for both clinical and economic success (Table 5). These implementations typically featured 2-way, high-definition audiovisual systems connecting EDs to remote specialists. Hub-and-spoke models were common, with specialist coverage ratios of 1:7-8 peripheral sites as documented by Demaerschalk et al [34] and Nelson et al [23]. Nelson et al [22] used a similar hub-and-spoke model with 2 hub facilities (1 primary stroke center and 1 comprehensive stroke center) supporting 17 spoke facilities, demonstrating scalability of this approach.

Other virtual modalities included mobile electrocardiogram transmission [8], telephone consultation with medication delivery [36], and a hybrid model combining 75% virtual with 25% in-person coverage [41]. Technology sophistication varied considerably, from basic telephone systems in resource-limited Haiti to advanced platforms with peripheral examination devices and rapid imaging transfer capabilities. Lower-technology solutions proved viable in resource-constrained settings. The Haiti telephone-based model [36] achieved cost-effectiveness despite, or perhaps because of, its simplicity, suggesting technology should match context rather than maximize sophistication. The in-ambulance telemedicine model [35] represents an intermediate technology approach, combining real-time consultation with automated data transmission. This prehospital intervention uniquely demonstrated that time gains as small as 6 minutes could achieve cost-effectiveness, with dominance achieved at 12 minutes.

**Table 5:** Virtual care modality comparison—types, cost-effectiveness, and implementation contexts.^a^

Virtual care modality	Technology platform	Clinical applications	Cost-effectiveness findings	Implementation setting	Key success factors	Challenges/limitations
Real-time video consultation	2-way HD^b^ videoconferencing Mobile carts with cameras Peripheral examination devices	Stroke [23,34,35,42] Pediatric emergencies [39,40,43] Neuroemergencies [37,38] Psychiatric emergencies [40]	3 of 4 studies indicate the dominant cost-saving of the intervention CAD $64.61 per YLL^c^ averted; Saves US $4662 per child/ED^d^/year € 365 per transfer avoided; 0-5000 AUD/QALY^e^ Lower charges; shorter LOS^f^ for the intervention	Rural EDs to urban specialists Hub-and-spoke networks 24/7 availability	Specialist expertise access Rapid decision-making Visual assessment capability High clinician satisfaction	Technology infrastructure costs Internet connectivity in rural areas Staff training requirements
Mobile ECG^g^ transmission	12-lead ECG recorder Mobile phone transmission Central hub interpretation	Acute coronary syndrome Cardiac arrhythmias Prehospital triage [33]	€990-€2508/QALY €16.70 vs €24.80-€55.20/ECG Lives saved for STEMI^h^ patients	Prehospital EMS^i^ units Regional coverage (4M population) 109,750 ECGs/year	Rapid cardiac diagnosis Avoided unnecessary transports 24/7 cardiologist availability	Limited to cardiac conditions Requires EMS training Mobile network dependency
Telephone consultation	Call center platform Twilio intake system Beacon driver dispatch	Pediatric preemergencies Nighttime service Medication delivery [36]	US $1288/DALY^j^ averted Cost-effective below 3× GDP 199.76 DALYs averted/year	Haiti (resource-limited) Nighttime-only service Home medication delivery	Addresses access barriers Low technology requirements Integrated medication delivery	Limited to preemergency cases No visual assessment Requires delivery infrastructure
Hybrid virtual-physical model	HD videoconferencing Wireless telehealth carts 75% virtual, 25% in-person	General ED presentations Rural hospital coverage predominantly lower acuity [41]	Dominant strategy AUD $105 savings/ED encounter Improved quality metrics	29 rural hospitals 24/7 coverage Virtual rural generalists	Workforce sustainability Reduced locum dependency Maintained care continuity	Requires some physical presence Complex scheduling Multiple site coordination

^a^Costs reported in non-US currencies in this table were converted to US dollars using the average annual exchange rate for the currency and cost year reported in each study: €1=US $1.39 (2009); €1=US $1.29 (2012); AUD $1=US $0.75 (2018); AUD $1=US $0.69 (2022); CAD $1=US $0.77 (2022). The same rates are applied throughout the manuscript.

^b^HD: high definition.

^c^YLL: years of life lost.

^d^ED: emergency department.

^e^QALY: quality-adjusted life year.

^f^LOS: length of stay.

^g^ECG: electrocardiogram.

^h^STEMI: ST-elevation myocardial infarction.

^i^EMS: emergency medical services.

^j^DALY: disability-adjusted life year.

#### By Provider Type

Provider types delivering virtual care included neurologists and stroke specialists [23,34,35,37,38,42], pediatric critical care physicians and psychiatrists [36,39,40,43], rural generalist physicians [41], cardiologists [8], and nurses with physician oversight [36]. Most programs operated 24/7, though the Haiti service functioned only during nighttime hours when traditional care was unavailable. A unique prehospital model was evaluated by Espinoza et al [35], featuring in-ambulance telemedicine with live bidirectional audio-video communication, automated vital signs transmission, and 24/7 neurologist consultation. This system served 150 patients per ambulance annually, with 39% of eligible patients receiving telemedicine consultation.

Sensitivity analyses were conducted in 12/13 (92%) studies. The most common approaches were 1-way deterministic sensitivity analysis (n=9), probabilistic sensitivity analysis using Monte Carlo simulation (n=7, with iterations ranging from 1000 to 10,000), 2-way sensitivity analysis (n=4), and scenario analysis (n=2). Kadel et al [37] were the only study that did not perform a sensitivity analysis. Key findings from sensitivity analyses included results were robust to parameter variation in 10/12 studies; Nelson et al [23] found that the ICER was most sensitive to the time horizon assumption, shifting from US $108,363/QALY at 90 days to US $2449/QALY over a lifetime; Nelson et al [22] demonstrated through probabilistic sensitivity analysis (10,000 iterations) that telestroke was the most cost-effective strategy in at least half of simulation runs when willingness-to-pay thresholds exceeded US $5000/QALY (spoke bearing 0% costs), US $30,000/QALY (50% costs), or US $52,000/QALY (100% costs); Espinoza et al [35] identified the minimum time gain threshold (6 minutes) needed for cost-effectiveness; and Yang et al [43] reported that ROI estimates remained favorable (range 1.28-1.96) across probabilistic iterations. Key uncertainty drivers across studies included time horizon, transfer rates, intervention use rates, cost-sharing arrangements, and discount rates. No study identified scenarios under which virtual care became definitively cost-ineffective in their primary sensitivity analyses, though shorter time horizons and lower use rates reduced the magnitude of economic benefits.

### Cost Components Analysis

Direct medical costs consistently favored virtual care, with per-consultation costs ranging from €16.70 (US $21) for electrocardiogram telemedicine [8] to US $600 per consultation for neurological telemedicine [42]. While initial infrastructure investment was substantial, break-even typically occurred within 1-3 years based on operational savings. The Victorian Stroke Telemedicine program showed cost savings of US $142 (AUD $190) per patient (control: US $61,631 [AUD $82,449]; intervention: US $61,489 [AUD $82,259]) at 12 months [38]. Direct medical costs consistently favored virtual care across multiple studies. Brunetti et al [8] reported telemedicine electrocardiogram consultation costs of €16.70 (US $21) per encounter compared to €24.80-€55.20 (US $32-71) for conventional ED visits in Italy. Similarly, Von Huben et al [41] documented cost savings of US $73 (AUD $105) per ED encounter for their Virtual Rural Generalist Service (US $93 vs US $167 [AUD $134 vs AUD $240] for usual care). Nelson et al [22] provided detailed cost breakdowns from POTN: spoke facility fixed costs included monthly camera fees (US $1980/month), 1-time equipment setup (US $11,000), and yearly training (US $768), while hub facility costs included 1-time equipment (US $26,300), annual wireless connectivity (US $4500), a full-time telestroke coordinator (US $89,679), and yearly training (US $900). These real-world cost data, derived from encounter-level financial records rather than literature estimates, represent a significant contribution to understanding the true infrastructure investment required for telestroke implementation.

Infrastructure investment varied considerably by program scope and technology. While initial setup costs could be substantial, rapid ROI was consistently demonstrated. Yang et al [43] calculated an ROI of 1.28 in their base case, rising to 1.96 in probabilistic analyses for pediatric telemedicine consultations.

Transfer and transportation represented the largest cost differential, particularly in rural settings. Virtual care programs achieved dramatic reductions in transfer rates—from 85%-90% to 5%-23% in patients with stroke [42] and a 73% reduction in neurosurgical transfers [37]. Nelson et al [22] reported a slight decline in transfer probability for tPA-treated patients (55.6% to 51.9%) but an increase among untreated patients (11.2% to 16.8%), the latter likely reflecting improved identification of patients requiring higher-level care. Beyond direct ambulance costs, virtual care eliminated family travel costs and productivity losses. The Canadian pediatric teleresuscitation program documented mean costs of US $1563 (CAD $2032.73) for telemedicine cases vs US $244 (CAD $317.45) for controls [39]. Transfer and transportation costs represented the largest area of savings. Whetten et al [42] reported cost savings of US $3729 per patient, primarily from reduced unnecessary transfers (85% to 5% for non-tPA patients and 90% to 23% for tPA patients). Kadel et al [37] documented a 73% reduction in transfer rates for neurosurgical emergencies, saving 139,916 km in travel distance and €3,095,863 (US $4,303,250) over 4 years. Beyond direct ambulance costs, virtual care eliminated associated family expenses and productivity losses.

Staffing efficiencies emerged as a significant economic driver. Hub-and-spoke models achieved specialist coverage ratios of 1:7-8 sites compared to traditional 1:1 models [23,34]. The POTN model [22] demonstrated a similar approach with 2 hub facilities supporting 17 spoke facilities, with an average of 14.4 patients with acute ischemic stroke per spoke per year. Von Huben et al [41] documented substantial reductions in expensive locum physician shifts (from 1456 to 609 days), with the Virtual Rural Generalist Service achieving a price per activity unit of AUD $1047 compared to AUD $1753 for usual care. Staffing efficiencies emerged as a critical economic driver. They reported a 60% reduction in expensive locum physician shifts from 1456 to 609 days annually, with the price per activity unit lower for virtual care (AUD $1047) compared to usual care (AUD $1753). Hub-and-spoke models achieved efficient specialist coverage, as demonstrated by Demaerschalk et al [34] with 1 hub hospital supporting 7 spoke hospitals.

The Haiti pediatric telemedicine program demonstrated unique cost-effectiveness at US $1288 per DALY averted, well below the World Health Organization cost-effectiveness threshold of 3 times per capita GDP (US $3531) [36]. This program included medication delivery, representing an innovative model combining consultation and pharmaceutical services.

### Implementation Factors

Studies consistently identified high initial technology costs as the primary implementation barrier, though rapid ROI mitigated this concern. Yang et al [43] demonstrated an ROI of 1.28 in base case analysis, with pediatric telemedicine generating annual cost-savings of US $46,620 per ED treating 10 children. Rural connectivity challenges were addressed through tiered technology approaches—the Haiti study successfully operated using basic telephone systems [36], while other programs required high-definition videoconferencing [41]. Technology infrastructure costs emerged as the primary implementation barrier across studies. However, flexibility in technology requirements facilitated adoption. Flaherty et al [36] successfully implemented a nighttime pediatric telemedicine service in Haiti using basic telephone technology, while Von Huben et al [41] used high-definition videoconferencing in Australian rural hospitals.

Facilitators of successful implementation included strong clinical champions, executive support, and phased rollout strategies targeting high-need populations. Programs focusing on time-critical conditions like stroke demonstrated early wins that facilitated expansion. The Victorian Stroke Telemedicine program strategically excluded 4 large hospitals with existing stroke protocols, focusing implementation where impact would be greatest [38]. Programs targeting high-need populations demonstrated early wins—stroke programs consistently showed increased tPA administration rates and improved outcomes [23,34].

Integration with existing workflows proved critical for success. Kadel et al [37] demonstrated efficiency with 75% of teleconsultations completed within 15 minutes and 90% within 30 minutes. The ACCESS program achieved consultation completion rates with neurologists available for rural EDs, resulting in cost savings of US $4241 per patient and QALY gains of 0.20 per patient [42]. Clinical workflow integration proved critical for success. Kadel et al [37] reported that 75% of teleneurosurgery consultations were completed within 15 minutes and 90% within 30 minutes, demonstrating efficiency that encouraged provider adoption. Similarly, Brunetti et al [8] achieved rapid electrocardiogram interpretation with remote cardiologists available 24/7.

Volume and case mix significantly influenced economic outcomes. The Brunetti et al study processed 109,750 electrocardiograms from 274,198 total emergency calls in 2012, achieving economies of scale with a cost per electrocardiogram/consultation of €16.70 (US $21) [8]. Programs with lower volumes, such as the Belgian in-ambulance telemedicine study with 39% use rates, still achieved cost-effectiveness when time gains exceeded 6-12 minutes [35].

Provider acceptance was enhanced by demonstrable benefits to work-life balance and patient outcomes. Thomas et al [40] reported reduced ED length of stay (5.5 vs 8.3 hours) and lower charges (US $3493 vs US $8611) for telepsychiatry compared to usual care. Pace et al [39] documented an ICER of US $50 (CAD $64.61) per year of life lost averted for pediatric teleresuscitation, demonstrating clear value.

Funding models and reimbursement structures varied by country and health care system. In the United States, several studies noted consultation fees ranging from US $600 per consult [42] to operational costs of US $222,800 annually for telepsychiatry services [40]. The dominant finding across studies was that virtual emergency care programs generated net savings despite initial investments.

## Discussion

### Principal Findings

This systematic review found that virtual emergency care was associated with favorable economic outcomes across diverse clinical applications and health care settings. While these findings suggest economic viability, the predominance of model-based evaluations (7/13, 54% of included studies) and the absence of empirical negative findings warrant cautious interpretation. Model-based analyses rely on assumptions about clinical pathways, costs, and transition probabilities that may not fully capture real-world complexities. Multiple mechanisms underlie these favorable outcomes, including avoiding patient transfers and improving operational efficiency. Our results present a supportive economic case beyond direct cost savings, highlighting workforce sustainability benefits as well. For instance, one study documented a 60% reduction in the use of costly locum physicians [41].

It is important to recognize that the clinical heterogeneity across included studies spanning telestroke, pediatric telemedicine, psychiatric emergencies, cardiac triage, and rural generalist services limits the generalizability of aggregate conclusions. Each clinical domain involves distinct patient populations, care pathways, cost structures, and outcome measures (QALYs, DALYs, transfers avoided, and length of stay). While the overall direction of findings was consistently positive, the evidence base is strongest for stroke care (supported by multiple studies with concordant findings) and more preliminary for psychiatric and general emergency applications (each supported by single studies).

### Comparison With Prior Work

Our findings extend earlier domain-specific reviews in several important ways. Previous systematic reviews of telestroke cost-effectiveness [7] have demonstrated favorable outcomes in stroke-specific applications, while reviews of telemedicine in emergency settings [7,17,18] have primarily focused on clinical effectiveness rather than economic outcomes. Our review is the first to synthesize economic evidence across the full spectrum of virtual emergency care modalities, demonstrating that the favorable economics observed in telestroke extend to pediatric, psychiatric, and general emergency applications. This broader scope strengthens the evidence base for policy decisions that span multiple clinical domains.

Unlike the mixed results reported for telemedicine cost-effectiveness in nonemergency settings by Whitten et al [45], our findings were uniformly positive—a discrepancy that may reflect emergency care’s unique characteristics (high baseline costs, time-sensitive decisions, and clear outcome metrics) rather than a true absence of unfavorable results. Furthermore, the success of simpler technologies in resource-constrained settings challenges the assumption that high technological sophistication is necessary for cost-effectiveness. For example, a telephone-based consultation model in Haiti was found to be cost-effective [36], suggesting that matching the technology to the context can be more important than using the most advanced tools. Our review also highlights the prehospital telemedicine model as an important middle ground: ambulance-based systems can achieve cost-effectiveness without a full hospital infrastructure.

Moreover, by including full economic evaluations rather than cost-only studies, our review provides more robust evidence than prior reviews that examined only cost data without health outcome integration.

### Interpretation of Cost-Effectiveness Findings

The cost-effectiveness of virtual emergency care was driven by distinct mechanisms across clinical domains. In stroke care, the primary value proposition was enabling time-sensitive thrombolysis through rapid specialist consultation, where even modest improvements in treatment times translated into substantial QALY gains and reduced long-term disability costs. The strong lifetime cost-effectiveness (ICER of US $2449/QALY) despite unfavorable short-term economics (US $108,363/QALY at 90 days) underscores the importance of capturing downstream benefits in economic evaluations of acute interventions.

In pediatric applications, cost-effectiveness was primarily driven by transfer avoidance and reduced unnecessary hospitalizations, reflecting the high baseline costs of pediatric emergency transfers and the ability of specialist teleconsultation to safely manage patients locally. The variability in ICERs across studies—ranging from US $1273 (€990)/QALY to US $108,363/QALY—reflects differences in time horizons, health care system costs, comparator practices, and the specific clinical conditions studied. These contextual factors mean that the transferability of specific ICER values to new settings requires careful consideration of local cost structures, existing care pathways, and patient populations.

### Policy and Clinical Implications

The evidence supports consideration of expanding virtual emergency care programs, particularly in settings where the economic case is strongest, while acknowledging that implementation decisions should account for local contextual factors not fully captured in economic models. The rapid uptake of virtual care during temporary COVID-19 policy relaxations demonstrated the feasibility of swift implementation. Health care administrators should prioritize high-impact virtual applications (eg, telestroke and pediatric critical care) in underserved areas to maximize early ROI and build organizational support; documented break-even periods of 12-24 months can reassure budget-conscious decision-makers.

Moreover, the fact that all economic perspectives examined showed positive results further strengthens the investment case: even analyses using a narrow health care system perspective demonstrated cost savings, while broader societal perspective analyses revealed added benefits such as reduced costs for families and productivity gains. For clinicians, it is significant that the economic benefits of virtual emergency care were accompanied by quality improvements (maintained or improved patient outcomes, >90% patient satisfaction, and reduced ED length of stay). This finding suggests that virtual care can enhance care quality rather than compromise it, while also reducing clinicians’ workload through improved patient flow.

### Methodological Considerations

Although the included studies used diverse economic evaluation methods reflecting real-world variability, this heterogeneity complicates direct comparisons and underscores the need for more standardized reporting of cost-effectiveness outcomes (eg, consistent use of ICER calculations, uniform costing methods, and common time horizons). The development of a core set of economic outcome measures for virtual emergency care could significantly improve comparability across studies.

While 54% (7/13) of the studies were model-based analyses, thereby raising some questions about generalizability, the consistency of findings between these models and the observational studies is reassuring. However, the reliance on modeling introduces inherent uncertainty, as results depend on assumptions about clinical pathways, transition probabilities, and cost structures that may not hold across all settings.

Overall, the quality of the economic evidence was generally high: on average, studies met over 90% of criteria on validated quality assessment checklists, though we note that these percentage scores should be interpreted as descriptive indicators rather than precise quality metrics, as these checklists were not designed for numerical aggregation. However, future evaluations should strengthen their reporting of generalizability and better disclose any conflicts of interest. Encouragingly, 92% (12/13) of the studies included sensitivity analyses, indicating that uncertainty was appropriately handled—which is an important practice for decision-maker confidence.

### Sensitivity Analysis and Uncertainty

While 92% (12/13) of included studies conducted sensitivity analyses, several important sources of uncertainty merit discussion. Key parameters driving variation in cost-effectiveness estimates included the time horizon of analysis, with shorter horizons (eg, 90 days) producing less favorable ICERs than lifetime models; patient volume and use rates, which influenced the spread of fixed infrastructure costs; transfer rates and distances, which were the dominant cost drivers in rural settings; and discount rates applied to future health benefits.

Notably, no study systematically explored scenarios in which virtual care would not be cost-effective, such as settings with very low patient volume, high existing specialist availability, or unreliable technology infrastructure. Future evaluations should explicitly model conditions under which virtual emergency care may not represent good value for money, to provide decision-makers with a more complete picture of when implementation is and is not warranted.

### Equity and Global Generalizability

The evidence base is dominated by high-income countries (the United States, Australia, Canada, Italy, and Belgium), with only one study conducted in a low-income setting (Haiti). This geographic concentration significantly limits the generalizability of findings to low- and middle-income countries (LMIC), where health care infrastructure, digital connectivity, workforce composition, and cost structures differ substantially. While the Haiti study demonstrated that simpler technologies can achieve cost-effectiveness in resource-constrained environments, this single study cannot address the diverse challenges faced across LMIC. Claims about global scalability should therefore be interpreted with caution.

Future research should prioritize economic evaluations in LMIC, where the potential impact of virtual emergency care may be greatest but where implementation barriers—including limited broadband access, electricity reliability, and trained workforce availability—are also most pronounced. Equity considerations, including whether virtual emergency care reduces or exacerbates disparities related to digital literacy, language, age, or socioeconomic status, also require dedicated investigation.

### Strengths and Limitations

This review implemented several measures to minimize bias. We conducted a comprehensive search across multiple databases, including gray literature and non-English studies, and applied rigorous quality assessments using validated tools alongside the GRADE approach. Furthermore, by focusing exclusively on full economic evaluations, we ensured that our conclusions reflect true value generation rather than cost shifting between settings.

Despite these strengths, the study has several limitations. The interventions evaluated were heterogeneous, which precluded a meaningful meta-analysis. In addition, most included studies adopted a health care system (rather than societal) perspective, potentially underestimating the full benefits of virtual care.

Publication bias represents a significant concern in this review. Despite our comprehensive search across 8 databases and gray literature sources, all 13 included studies reported favorable economic outcomes for virtual emergency care. The complete absence of negative or neutral findings is noteworthy and may reflect selective publication of studies with positive results, selective reporting of favorable outcomes within studies, or genuine cost-effectiveness of virtual emergency care across all evaluated contexts. The possibility that unfavorable economic evaluations remain unpublished or were not identified by our search cannot be excluded. This limitation should temper the certainty with which the overall positive conclusions are interpreted.

Several important cost dimensions are underrepresented in the included studies. Ongoing technology maintenance and upgrade costs, which can be substantial as hardware ages and software requires updates, were rarely incorporated. Staff training costs—both initial and recurrent—were inconsistently reported, yet represent a significant and recurring investment. Workflow integration costs, including the time and resources required to redesign clinical processes, develop protocols, and manage change, were largely absent from economic evaluations. Additionally, the included studies did not systematically account for potential downstream costs such as follow-up care resulting from remote diagnostic limitations, liability and malpractice considerations, or the costs of addressing technology failures and downtime.

The long-term sustainability of cost savings remains uncertain, as initial efficiencies may diminish as programs scale, technology costs evolve, or reimbursement structures change. While early evidence indicates that the benefits are sustained or even amplified over time, longer-term studies will be important to confirm this durability.

While this review adhered to PRISMA 2020 guidelines, certain recommended items—including formal assessment of reporting bias across studies and provision of underlying extracted data—were not fully addressed. The complete dataset of extracted study characteristics and economic outcomes is available from the corresponding author upon request.

### Future Research Directions

Looking ahead, further research is needed in several key areas. First, economic evaluation methods should be standardized to facilitate cross-study comparisons and enable future meta-analyses. Second, researchers should evaluate emerging virtual care modalities such as AI-driven triage algorithms and integrated digital emergency systems to determine which features truly drive cost-effectiveness and which simply add expense. Third, dedicated studies should assess the equity impacts of virtual emergency care to determine whether it reduces or exacerbates health care disparities, with particular attention to the digital divide. Fourth, implementation science approaches are needed to optimize how virtual emergency care is delivered. Comparative studies of different care models, training methods, and change management strategies could offer practical guidance for health systems, given that current variation in program success suggests significant room for improvement. Finally, long-term evaluations are crucial to assess how the benefits of virtual emergency care evolve over time, how quality of care is maintained as programs scale up, and how these models adapt to changing technology landscapes.

Future evaluations should also adopt longer time horizons and incorporate comprehensive implementation cost frameworks—including technology maintenance, staff training, workflow redesign, and opportunity costs—to provide a more complete economic picture. Studies should explicitly model conditions under which virtual emergency care may not be cost-effective to help decision-makers identify appropriate implementation contexts.

Despite these research needs, the existing evidence supports broader implementation of virtual emergency care, particularly for high-need populations and time-sensitive emergency conditions where its benefits are most pronounced.

### Conclusion

This systematic review found consistent evidence of favorable economic outcomes for virtual emergency care across diverse clinical conditions, settings, and modalities. The evidence is strongest for stroke care and rural/remote implementations, where transfer avoidance drives substantial savings. While all included studies reported cost-effectiveness or cost savings, the predominance of model-based analyses, the absence of evidence from LMIC, and the possibility of publication bias warrant cautious interpretation. These findings support the continued expansion of virtual emergency care, particularly for high-need populations and time-sensitive conditions, while highlighting the need for standardized economic evaluation methods, long-term sustainability assessments, and equity-focused research to guide future implementation decisions.

## Appendix

Multimedia Appendix 1 PRISMA checklist.

Multimedia Appendix 2 Full electronic search strategies for all eight databases.

Multimedia Appendix 3 Item-level methodological quality assessment of the included studies using the Drummond and CHEC checklists.

## Abbreviations

ACCESS: Access to Critical Cerebral Emergency Support Services
CHEC: Consensus Health Economic Criteria
DALY: disability-adjusted life year
ED: emergency department
GRADE: Grading of Recommendations Assessment, Development, and Evaluation
ICER: incremental cost-effectiveness ratio
LMIC: low- and middle-income countries
NIHSS: National Institutes of Health Stroke Scale
POTN: Providence Oregon Telestroke Network
PRISMA: Preferred Reporting Items for Systematic Reviews and Meta-Analyses
PROSPERO: International Prospective Register of Systematic Reviews
QALY: quality-adjusted life year
ROI: return on investment
tPA: tissue plasminogen activator

## Data Availability

All data generated or analyzed during this study are included in this published article and its supplementary information files. The complete search strategy, data extraction forms, and quality assessment ratings are available in Multimedia Appendices 2 and 3. The dataset of extracted study characteristics and economic outcomes is available from the corresponding author upon reasonable request.
