# Supplementary material for: Cost-Effectiveness of Virtual Emergency Care Models: Systematic Review
Source: JMIR Mhealth Uhealth. 2026 Aug 5;14:e82143. doi: 10.2196/82143 (PMC13490934; doi:10.2196/82143)
Supplement: Supplementary file 2 [file mhealth_v14i1e82143_app2.docx]

**Multimedia Appendix 1: Database Search Strategies**

**Search Period:** Database inception to February 2025

**1. PubMed/MEDLINE (n=2,084)**

((virtual[tiab] OR telehealth[tiab] OR telemedicine[mesh] OR telemedicine[tiab] OR digital[tiab] OR "remote consult*"[tiab] OR mHealth[tiab] OR eHealth[tiab] OR "mobile health"[tiab] OR "web-based"[tiab] OR wearable*[tiab] OR "remote monitoring"[tiab])

AND

(emergency[tiab] OR emergencies[tiab] OR "emergency department"[mesh] OR "emergency department"[tiab] OR ED[tiab] OR ER[tiab] OR "emergency room"[tiab] OR trauma[tiab] OR "critical care"[tiab])

AND

("cost effectiv*"[tiab] OR "cost utility"[tiab] OR "cost benefit"[tiab] OR "cost minimization"[tiab] OR "economic evaluation"[tiab] OR "cost analysis"[tiab] OR "return on investment"[tiab] OR ICER[tiab] OR QALY[tiab]))

**2. Embase (n=1,827)**

('virtual care'/exp OR 'telehealth'/exp OR 'telemedicine'/exp OR 'digital health'/exp OR 'remote consultation'/exp OR 'mobile health'/exp OR 'ehealth'/exp OR 'wearable device'/exp OR 'remote monitoring'/exp OR virtual:ti,ab OR telehealth:ti,ab OR telemedicine:ti,ab OR digital:ti,ab OR 'remote consult*':ti,ab OR mhealth:ti,ab OR ehealth:ti,ab OR 'mobile health':ti,ab OR 'web-based':ti,ab OR wearable*:ti,ab OR 'remote monitoring':ti,ab)

AND

('emergency ward'/exp OR 'emergency medicine'/exp OR 'trauma'/exp OR 'critical care'/exp OR emergency:ti,ab OR emergencies:ti,ab OR 'emergency department':ti,ab OR ed:ti,ab OR er:ti,ab OR 'emergency room':ti,ab OR trauma:ti,ab OR 'critical care':ti,ab)

AND

('cost effectiveness analysis'/exp OR 'cost utility analysis'/exp OR 'cost benefit analysis'/exp OR 'cost minimization analysis'/exp OR 'economic evaluation'/exp OR 'cost effectiv*':ti,ab OR 'cost utility':ti,ab OR 'cost benefit':ti,ab OR 'cost minimization':ti,ab OR 'economic evaluation':ti,ab OR 'cost analysis':ti,ab OR 'return on investment':ti,ab OR icer:ti,ab OR qaly:ti,ab)

**3. Scopus (n=916)**

(TITLE-ABS-KEY(virtual OR telehealth OR telemedicine OR digital OR "remote consult*" OR mhealth OR ehealth OR "mobile health" OR "web-based" OR wearable* OR "remote monitoring"))

AND

(TITLE-ABS-KEY(emergency OR emergencies OR "emergency department" OR ed OR er OR "emergency room" OR trauma OR "critical care"))

AND

(TITLE-ABS-KEY("cost effectiv*" OR "cost utility" OR "cost benefit" OR "cost minimization" OR "economic evaluation" OR "cost analysis" OR "return on investment" OR icer OR qaly))

**4. Web of Science (n=645)**

TS=((virtual OR telehealth OR telemedicine OR digital OR "remote consult*" OR mhealth OR ehealth OR "mobile health" OR "web-based" OR wearable* OR "remote monitoring")

AND

(emergency OR emergencies OR "emergency department" OR ed OR er OR "emergency room" OR trauma OR "critical care")

AND

("cost effectiv*" OR "cost utility" OR "cost benefit" OR "cost minimization" OR "economic evaluation" OR "cost analysis" OR "return on investment" OR icer OR qaly))

**5. CINAHL (n=199)**

(MH "Telemedicine+" OR MH "Telehealth+" OR MH "Mobile Health+" OR MH "Digital Health+" OR TI virtual OR AB virtual OR TI telehealth OR AB telehealth OR TI telemedicine OR AB telemedicine OR TI digital OR AB digital OR TI "remote consult*" OR AB "remote consult*" OR TI mhealth OR AB mhealth OR TI ehealth OR AB ehealth OR TI "mobile health" OR AB "mobile health" OR TI "web-based" OR AB "web-based" OR TI wearable* OR AB wearable* OR TI "remote monitoring" OR AB "remote monitoring")

AND

(MH "Emergency Service+" OR MH "Emergency Medicine+" OR MH "Trauma Centers+" OR TI emergency OR AB emergency OR TI emergencies OR AB emergencies OR TI "emergency department" OR AB "emergency department" OR TI ed OR AB ed OR TI er OR AB er OR TI "emergency room" OR AB "emergency room" OR TI trauma OR AB trauma OR TI "critical care" OR AB "critical care")

AND

(MH "Economics+" OR MH "Cost-Benefit Analysis+" OR TI "cost effectiv*" OR AB "cost effectiv*" OR TI "cost utility" OR AB "cost utility" OR TI "cost benefit" OR AB "cost benefit" OR TI "cost minimization" OR AB "cost minimization" OR TI "economic evaluation" OR AB "economic evaluation" OR TI "cost analysis" OR AB "cost analysis" OR TI "return on investment" OR AB "return on investment" OR TI icer OR AB icer OR TI qaly OR AB qaly)

**6. Cochrane Library (n=82)**

#1 MeSH descriptor: [Telemedicine] explode all trees

#2 MeSH descriptor: [Mobile Health Units] explode all trees

#3 (virtual OR telehealth OR telemedicine OR digital OR "remote consult*" OR mhealth OR ehealth OR "mobile health" OR "web-based" OR wearable* OR "remote monitoring"):ti,ab,kw

#4 #1 OR #2 OR #3

#5 MeSH descriptor: [Emergency Service, Hospital] explode all trees

#6 MeSH descriptor: [Emergency Medicine] explode all trees

#7 (emergency OR emergencies OR "emergency department" OR ed OR er OR "emergency room" OR trauma OR "critical care"):ti,ab,kw

#8 #5 OR #6 OR #7

#9 MeSH descriptor: [Economics] explode all trees

#10 MeSH descriptor: [Cost-Benefit Analysis] explode all trees

#11 ("cost effectiv*" OR "cost utility" OR "cost benefit" OR "cost minimization" OR "economic evaluation" OR "cost analysis" OR "return on investment" OR icer OR qaly):ti,ab,kw

#12 #9 OR #10 OR #11

#13 #4 AND #8 AND #12

**7. MEDLINE (via Ovid) (n=30)**

1. exp Telemedicine/

2. exp Mobile Health Units/

3. (virtual or telehealth or telemedicine or digital or "remote consult*" or mhealth or ehealth or "mobile health" or "web-based" or wearable* or "remote monitoring").ti,ab.

4. 1 or 2 or 3

5. exp Emergency Service, Hospital/

6. exp Emergency Medicine/

7. (emergency or emergencies or "emergency department" or ed or er or "emergency room" or trauma or "critical care").ti,ab.

8. 5 or 6 or 7

9. exp Economics/

10. exp Cost-Benefit Analysis/

11. ("cost effectiv*" or "cost utility" or "cost benefit" or "cost minimization" or "economic evaluation" or "cost analysis" or "return on investment" or icer or qaly).ti,ab.

12. 9 or 10 or 11

13. 4 and 8 and 12

**8. PsycINFO (n=3)**

1. DE "Telemedicine" OR DE "Telehealth"

2. TI (virtual OR telehealth OR telemedicine OR digital OR "remote consult*" OR mhealth OR ehealth OR "mobile health" OR "web-based" OR wearable* OR "remote monitoring") OR AB (virtual OR telehealth OR telemedicine OR digital OR "remote consult*" OR mhealth OR ehealth OR "mobile health" OR "web-based" OR wearable* OR "remote monitoring")

3. 1 OR 2

4. DE "Emergency Services" OR DE "Crisis Intervention"

5. TI (emergency OR emergencies OR "emergency department" OR ed OR er OR "emergency room" OR trauma OR "critical care") OR AB (emergency OR emergencies OR "emergency department" OR ed OR er OR "emergency room" OR trauma OR "critical care")

6. 4 OR 5

7. DE "Cost Effectiveness" OR DE "Costs and Cost Analysis"

8. TI ("cost effectiv*" OR "cost utility" OR "cost benefit" OR "cost minimization" OR "economic evaluation" OR "cost analysis" OR "return on investment" OR icer OR qaly) OR AB ("cost effectiv*" OR "cost utility" OR "cost benefit" OR "cost minimization" OR "economic evaluation" OR "cost analysis" OR "return on investment" OR icer OR qaly)

9. 7 OR 8

10. 3 AND 6 AND 9

**Additional Sources**

**Grey Literature Sources (n=17)**

- OpenGrey
- Google Scholar (first 200 results)
- ProQuest Dissertations & Theses Global
- Health Technology Assessment Database
- NHS Economic Evaluation Database
- Government websites (CDC, NHS, Health Canada, etc.)
- Professional organization reports

**Citation Searching (n=14)**

- Forward and backward citation searching of included studies
- Reference lists of relevant systematic reviews
- Key author searches

**Search Filters and Limitations**

**Language:** No language restrictions applied
**Publication Type:** No restrictions on study design
**Date:** No date restrictions (inception to February 2025)
**Publication Status:** Published and unpublished studies included

**Search Validation**

The search strategy was validated by:

1. Ensuring retrieval of known relevant studies
2. Expert review by information specialist
3. Pilot testing across databases
4. Iterative refinement based on initial results

**Total unique references after deduplication:** 5,786
**Additional references from citation/grey literature searching:** 31
