# Supplementary material for: Cost-Effectiveness of Virtual Emergency Care Models: Systematic Review
Source: JMIR Mhealth Uhealth. 2026 Aug 5;14:e82143. doi: 10.2196/82143 (PMC13490934; doi:10.2196/82143)
Supplement: Supplementary file 3 [file mhealth_v14i1e82143_app3.docx]

**Multimedia Appendix 2:** Drummond-10 checklist

Q1: Was a well-defined question posed in answerable form?

Q2: Was a comprehensive description of the competing alternatives given?

Q3: Was the effectiveness of the programme or services established?

Q4: Were all the important and relevant costs and consequences for each alternative identified?

Q5: Were costs and consequences measured accurately in appropriate physical units?

Q6: Were the cost and consequences valued credibly?

Q7: Were costs and consequences adjusted for differential timing?

Q8: Was an incremental analysis of costs and consequences of alternatives performed?

Q9: Was allowance made for uncertainty in the estimates of costs and consequences?

Q10: Did the presentation and discussion of study results include all issues of concern to users?

| **First author** | **Q1** | **Q2** | **Q3** | **Q4** | **Q5** | **Q6** | **Q7** | **Q8** | **Q9** | **Q10** | **Score** |
| --- | --- | --- | --- | --- | --- | --- | --- | --- | --- | --- | --- |
| Brunetti, 2014 | Yes | Yes | Yes | Yes | Yes | Yes | No | Yes | No | Yes | 8.0 |
| Demaerschalk, 2013 | Yes | Yes | Yes | Yes | Yes | Yes | Yes | Yes | Yes | Yes | 10.0 |
| Espinoza, 2017 | Yes | Yes | Yes | Yes | Yes | Yes | Yes | Yes | Yes | Yes | 10.0 |
| Flaherty, 2022 | Yes | Yes | Yes | Yes | Yes | Yes | Partial | Yes | Yes | Yes | 9.5 |
| Kadel, 2018 | Yes | Yes | Yes | Yes | Yes | Yes | Yes | Yes | No | Yes | 9.0 |
| Kim, 2022 | Yes | Yes | Yes | Yes | Yes | Yes | Yes | Yes | Yes | Yes | 10.0 |
| Nelson, 2011 | Yes | Yes | Yes | Yes | Yes | Yes | Yes | Yes | Yes | Yes | 10.0 |
| Nelson, 2016 | Yes | Yes | Yes | Yes | Yes | Yes | No | Yes | Yes | Yes | 9.0 |
| Pace, 2023 | Yes | Yes | Yes | Yes | Yes | Yes | No | Yes | Yes | Yes | 9.0 |
| Thomas, 2018 | Yes | Yes | Yes | Yes | Yes | Yes | Yes | Yes | No | Yes | 9.0 |
| Von Huben, 2024 | Yes | Yes | Yes | Yes | Yes | Yes | No | Yes | Yes | Yes | 9.0 |
| Whetten, 2018 | Yes | Yes | Yes | Yes | Yes | Yes | No | Yes | Yes | Yes | 9.0 |
| Yang, 2015 | Yes | Yes | Yes | Yes | Yes | Yes | No | Yes | Yes | Yes | 9.0 |

**Supplementary Table S2:** CHEC-19 checklist

Q1: Is the study population clearly described?

Q2: Are competing alternatives clearly described?

Q3: Is a well-defined research question posed in answerable form?

Q4: Is the economic study design appropriate to the stated objective?

Q5: Is the chosen time horizon appropriate to include relevant costs and consequences?

Q6: Is the actual perspective chosen appropriate?

Q7: Are all important and relevant costs for each alternative identified?

Q8: Are all costs measured appropriately in physical units?

Q9: Are costs valued appropriately?

Q10: Are all important and relevant outcomes for each alternative identified?

Q11: Are all outcomes measured appropriately?

Q12: Are outcomes valued appropriately?

Q13: Is an incremental analysis of costs and outcomes of alternatives performed?

Q14: Are all future costs and outcomes discounted appropriately?

Q15: Are all important variables, whose values are uncertain, appropriately subjected to sensitivity analysis?

Q16: Do the conclusions follow from the data reported?

Q17: Does the study discuss the generalizability of the results to other settings and patient/client groups?

Q18: Does the article indicate that there is no potential conflict of interest of study researcher(s) and funder(s)?

Q19: Are ethical and distributional issues discussed appropriately?

| **First author** | **Q1** | **Q2** | **Q3** | **Q4** | **Q5** | **Q6** | **Q7** | **Q8** | **Q9** | **Q10** | **Q11** | **Q12** | **Q13** | **Q14** | **Q15** | **Q16** | **Q17** | **Q18** | **Q19** | **Score** |
| --- | --- | --- | --- | --- | --- | --- | --- | --- | --- | --- | --- | --- | --- | --- | --- | --- | --- | --- | --- | --- |
| Brunetti, 2014 | Yes | Yes | Yes | Yes | Yes | Yes | Yes | Yes | Yes | Yes | Yes | Yes | Yes | No | No | Yes | Partial | Yes | Partial | 16.0 |
| Demaerschalk, 2013 | Yes | Yes | Yes | Yes | Yes | Yes | Yes | Yes | Yes | Yes | Yes | Yes | Yes | Yes | Yes | Yes | Yes | Yes | Partial | 18.5 |
| Espinoza, 2017 | Partial | Yes | Yes | Yes | Yes | Yes | Yes | Yes | Yes | Yes | Yes | Yes | Yes | Yes | Yes | Yes | Yes | Partial | Partial | 17.5 |
| Flaherty, 2022 | Yes | Yes | Yes | Yes | Yes | Yes | Yes | Yes | Yes | Yes | Yes | Yes | Yes | Partial | Yes | Yes | Yes | Yes | Yes | 18.5 |
| Kadel, 2018 | Yes | Yes | Yes | Yes | Yes | Yes | Yes | Yes | Yes | Yes | Yes | Yes | Yes | Yes | Partial | Yes | Yes | Yes | Yes | 18.5 |
| Kim, 2022 | Yes | Yes | Yes | Yes | Yes | Yes | Yes | Yes | Yes | Yes | Yes | Yes | Yes | Yes | Yes | Yes | Yes | Yes | Yes | 19.0 |
| Nelson, 2011 | Yes | Yes | Yes | Yes | Yes | Yes | Yes | Yes | Yes | Yes | Yes | Yes | Yes | Yes | Yes | Yes | Yes | Yes | Partial | 18.5 |
| Nelson, 2016 | Yes | Yes | Yes | Yes | Partial | Yes | Yes | Yes | Yes | Yes | Yes | Yes | Yes | No | Yes | Yes | Partial | Yes | Partial | 17.0 |
| Pace, 2023 | Yes | Yes | Yes | Yes | Yes | Yes | Yes | Yes | Yes | Yes | Yes | Yes | Yes | Yes | Yes | Yes | Yes | Yes | Yes | 19.0 |
| Thomas, 2018 | Yes | Yes | Yes | Yes | Yes | Yes | Yes | Yes | Yes | Yes | Yes | Yes | Yes | Partial | No | Yes | Yes | Yes | Yes | 17.5 |
| Von Huben, 2024 | Yes | Yes | Yes | Yes | Yes | Yes | Yes | Yes | Yes | Yes | Yes | Yes | Yes | Partial | Yes | Yes | Yes | Yes | Yes | 18.5 |
| Whetten, 2018 | Yes | Yes | Yes | Yes | Yes | Yes | Yes | Yes | Yes | Yes | Yes | Yes | Yes | Yes | Yes | Yes | Partial | Yes | Partial | 18.0 |
| Yang, 2015 | Yes | Yes | Yes | Yes | Yes | Yes | Yes | Yes | Yes | Yes | Yes | Yes | Yes | Yes | Yes | Yes | Partial | Yes | Partial | 18.0 |
